# Identification of parental line specific effects of MLF2 on resistance to coccidiosis in chickens

**DOI:** 10.1186/1753-6561-5-S4-S21

**Published:** 2011-06-03

**Authors:** Yeong Ho Hong, Eui-Soo Kim, Hyun S Lillehoj

**Affiliations:** 1 Department of Animal Science and Technology, Chung-Ang University Gyeonggi-Do 456-756, Korea; 2 Bovine Functional Genomics Laboratory, Animal and Natural Resources Institute, United States Department of Agriculture, Beltsville, MD 20705, USA; 3 Animal Parasitic Diseases Laboratory, Animal and Natural Resources Institute, United States Department of Agriculture, Beltsville, MD 20705, USA

## Abstract

**Background:**

*MLF2* was the candidate gene associated with coccidiosis resistance in chickens. Although single marker analysis supported the association between *MLF2* and coccidiosis resistance, causative mutation relevant to coccidiosis was not identified yet. Thus, this study suggested segregation analysis of *MLF2* haplotype and the association test of the other candidate genes using improved data transformation.

**Results:**

A haplotype probably originated from one parental line was found out of 4 major haplotypes of *MLF2*. Frequency of this haplotype was 0.2 in parental chickens and its offspring in 12 families. Allele substitution effect of the *MLF2* haplotype originated from a specific line was associated with increased body weight and fecal egg count explaining coccidiosis resistance. Nevertheless Box-Cox transformation was able to improve normality; association test did not produce obvious different results compared with analysis with log transformed phenotype.

**Conclusion:**

Allele substitution effect analysis and classification of *MLF2* haplotype identified the segregation of haplotype associated with coccidiosis resistance. The haplotype originated from a specific parental line was associated with improving disease resistance. Estimating effect of *MLF2* haplotype on coccidiosis resistance will provide useful information for selecting animals or lines for future study.

## Background

Avian coccidiosis impairs the growth and feed efficiency of infected chickens [1]. There were evidences that resistance to avian coccidiosis is associated with inheritance and coccidiosis resistant line could be established by selection [2]. The resistance to avian coccidiosis QTL has been identified near two microsatellite markers on chromosome 1 [3,4]. Associations between parameters of resistance to coccidiosis and single nucleotide polymorphisms (SNPs) in 3 candidate genes located around QTL on chromosome 1 (*Zyxin*, *TCR-β*, *MLF2*) were determined [5,6]. These studies showed the SNPs in *MLF2* are the most probable locus associated with coccidiosis resistance in chickens. The current analysis was conducted to identify the parental line specific haplotype of *MLF2*. Although *MLF2* explained variation of body weight affected by coccidiosis, the association of causative mutation was not obviously identified [6]. Thus, this study was suggested to identify segregation of *MLF2* allele associated with coccidiosis resistance for practical application and future study for identifying causative mutation. Since oocyst shedding significantly deviated from normal distribution, transformation method may affect association test. In previous studies, oocyst shedding was transformed using log transformation, but normality was fully satisfied (*p* ~ 0.01) by log transformation compared with Box-Cox transformation (*p* > 0.05). Box-Cox transformation of oocyst shedding was used to evaluate association between oocyst shedding and SNPs, including candidate genes analyzed in previous studies.

## Methods

Chickens from coccidiosis resistant and susceptible commercial broiler lines were crossed to produce F_1_ chickens [3]. Twelve pairs of F_1_ individuals were mated to produce 290 F_2_ offspring from four hatches. F_2_ chickens were orally infected with 1.0 x 10^4^ of sporulated *E. maxima* oocysts at 4 weeks of age [3]. Body weight was measured on days 3, 6, and 9 post-infection (PI) as previously described [7,8]. The number of oocysts was evaluated using the fecal sample collected between Days 5 to 9 PI by method of Lillehoj and Ruff (1987).

Genomic DNA was extracted from erythrocytes using the GenElute Blood Genomic DNA kit (Sigma, St. Louis, MO). PCR product was sequenced with internal primers using an ABI 3730 DNA analyzer (Applied Biosystem, Foster City, CA). SNP was genotyped by genomic DNA sequencing with the same gene-specific internal primers [5,6]. Then, haplotype phase was decided based on pedigree information. Associations between SNP genotypes or haplotypes and disease phenotypes were evaluated using linear model including family, sex, and hatch effects [5]. The number of oocyst was transformed by log transformation, and it was also transformed using an empirical approach suggested by Box-Cox [9]. These methods use the profile likelihood function for the largest linear model to be considered as a guide in choosing a value for parameter [10].

## Results

Ten SNPs were identified in the *zyxin*, 12 in the *TCR-β* and 4 in the *MLF2* gene [5,6]. At various times following experimental infection of the F_2_ generation with *Eimeria maxima*, body weights, fecal oocyst shedding, and biochemical parameters were measured as parameters of coccidiosis resistance [3]. Single marker and haplotype-based tests were applied to determine the associations between SNPs and the parameters of coccidiosis resistance including body weight and Box-Cox transformed oocyst shedding. The maximum additive genetic effect on disease resistance of a SNP in *MLF2* was explained by body weight (*p* = 0.0002) and this SNP of *MLF2* also significantly associated with body weight was also associated with fecal oocyst shedding [6]. This was confirmed using Box-Cox transformed oocyst shedding data in this study. Box-Cox transformed fecal oocyst shedding was close to normal distribution (Kolmogorov-Smirnov normality test, *p* > 0.1) compared with log transformed data (*p* > 0.01). Associations between Box-Cox transformed oocyst shedding number and SNP was summarized in Table 1. This analysis support evidence of association between markers in *MLF2* gene and oocyst shedding. Association between transformed oocyst shedding and the other genes including *zyxin* /*TCR-β* were also detected (*p* < 0.05) using Box-Cox transformed oocyst shedding, which was not significant using log transformed data. The significance level of association between the other markers and oocyst shedding was not changed obviously at various thresholds (*p* = 0.05, 0.01 or 0.001) compared with analysis using log transformed oocyst shedding.

**Table 1:** Association test using oocyst shedding transformed by Box-Cox transformation and log transformation

SNP	Log transformation	Box-Cox transformation
^1^*zyxin* SNP_187	0.08	0.04
^2^*TCR-β* SNP_177	0.06	0.02
^2^*MLF2-*SNP_892	0.002	0.001
^2^*MLF2-*SNP_947	0.02	0.02

^1^[5]

^2^[6]

Four haplotypes accounted for 98% of all observed *MLF2* SNP (Table 2). The haplotype association test was used to determine the relationship between the oocyst shedding number and the haplotypes of the *MLF2*. The allele substitution effect of *MLF2* haplotype 4 versus haplotypes 1 and 3 was significantly associated with increased body weight and oocyst shedding number (Table 2). The *MLF2* homozygous haplotypes 2/2 and 4/4 were found in 2 and 4 animals, respectively (Tables 3, 4). However, homozygous haplotypes 1 and 3 were not observed in 24 parent chickens, which implies that only haplotypes 1 (n=4) and 3 (n=10) were possibly originated from a specific parental chicken line. The haplotype substitution effect of haplotype 3 was higher than allele substitution effect of haplotypes 1 and 2 (Tables 3, 4). Haplotypes 2 and 4 were found in both parental line, but haplotypes 1 and 3 were likely to be originated from specific parental line. However, haplotypes 1 and 3 were likely to be originated from different parental chicken lines considering high heterozygous frequency of haplotypes 1 and 3 (Table 3).

**Table 2:** Allele (haplotype) substation effect of MLF2

Haplotype	ID	Frequency	Body weight (s.d.)	Oocyst shedding (s.d.)
A A T A	1	0.12	88.8 (42.1)**	2.63 (0.05)*
A T T A	2	0.26	41.7 (31.4)	1.47 (0.17)
A T T G	3	0.20	112.0 (38.0)**	2.13 (0.07)^+^
G T T A	4	0.41	-	-
others	-	0.01	-	-

** p < 0.01, * p < 0.05, + p < 0.1

**Table 3:** MLF2 haplotype in 24 parental chickens (F1)

Haplotype*	id	Number in parents	Homozygote	Heterozygous with haplotype 3	Origin of haplotype
A T T A	1	4	0	2	one or both
A A T A	2	14	2	4	both
A T T G	3	10	0	-	one or both
G T T A	4	18	4	3	both
others	-	2	0	1	na

* [6]

**Table 4:** Haplotypes of MLF2 in 24 parental chickens

Haplotypes					
Faimly-ID	1	2	3	4	others
F1-1	1	1	0	0	0
F1-2	1	0	0	1	0
F2-1	1	0	1	0	0
F2-2	0	0	1	1	0
F3-1	0	1	0	1	0
F3-2	0	0	0	1	1
F4-1	0	1	1	0	0
F4-2	0	1	1	0	0
F5-1	0	1	0	1	0
F5-2	0	1	0	1	0
F6-1	0	0	1	1	0
F6-2	0	0	0	**2**	0
F7-1	0	**2**	0	0	0
F7-2	0	1	1	0	0
F8-1	0	1	0	1	0
F8-2	0	0	0	**2**	0
F9-1	0	0	1	0	1
F9-2	0	0	0	**2**	0
F10-1	0	1	0	1	0
F10-2	0	0	1	1	0
F11-1	0	1	1	0	0
F11-2	0	0	0	**2**	0
F12-1	0	**2**	0	0	0
F12-2	1	0	1	0	0

## Conclusions

Single marker association analysis for 16 traits of coccidiosis resistance showed SNPs of *TCR-β* and *MLF2* were associated with oocyst shedding and body weights in previous study [6], and it was confirmed by additional association tests in current study. In this study allele substitution effect analysis and classification of *MLF2* haplotype elucidated parental origin of haplotype associated with coccidiosis resistance. The fourth [A/G] SNP in *MLF2* was major determinant of haplotype 3 vs the other haplotypes, but allele A of the first SNP [A/G] was common in haplotypes 1, 2 and 3. Haplotype association test was applied to estimate effect of each haplotype which was not revealed clearly by single marker association tests. Difference between additive effect (body weight and oocyst) of haplotypes 1 and 3 was smaller than that of haplotypes 2 and 3. However, haplotypes 1 and 3 are likely to be originated from the different parental lines. In genome-wide [3] and fine mapping [4] of coccidiosis resistance, QTL was not detected in some chicken families. These previous linkage QTL mapping studies could be affected by heterogeneity in parental lines. The haplotype (ATTG) of *MLF2* originated from a specific parental line was associated with elevated body weight and oocyst shedding number. Identifying haplotype diversity of *MLF2* in other chicken families will provide useful information for experimental designs in the future studies.

## List of abbreviations used

SNPs: single nucleotide polymorphisms; *MLF2:* Myeloid Leukemia Factor 2

## Competing interests

The authors declare that they have no competing interests.

## Authors’ contribution

YHH; genotyping and manuscript preparation, ESK; genetic analysis, HSL; grant preparation and experiment design
